# Large-scale research on durability test cycle of fuel cell system based on CATC

**DOI:** 10.1038/s41598-024-59536-z

**Published:** 2024-04-18

**Authors:** Hao Lan, Dong Hao, Zhiyang Su, Tianlei Zheng, Shaohui Liu, Jicheng Ma, Yuntang He, Lei Gao, Zhao Wang

**Affiliations:** 1 China Automotive Technology and Research Center Co., Ltd., Tianjin, 300300 China; 2 CATARC New Energy Vehicle Test Center (Tianjin) Co., Ltd., Tianjin, 300300 China; 3 SHANGHAI REFIRE Group Co., Ltd., Shanghai, 201803 China

**Keywords:** Fuel cell system durability, Test cycle, Model and validation, Hydrology, Engineering

## Abstract

Durability is one of the technical bottlenecks restricting fuel cell electric vehicle development. As a result, significant time and resources have been invested in research related to this area worldwide. Current durability research mainly focuses on the single cell and stack levels, which is quite different from the usage scenarios of actual vehicles. There is almost no research on developing durability test cycles on the fuel cell system level. This paper proposes a universal model for developing a durability test cycle for fuel cell system based on the China automotive test cycle. Large-scale comparison tests of the fuel cell systems are conducted. After 1000 h test, the output performance degradation of three mass-produced fuel cell system is 14.49%, 9.59%, and 4.21%, respectively. The test results show that the durability test cycle proposed in this paper can effectively accelerate the durability test of the fuel cell system and evaluate the durability performance of the fuel cell system. Moreover, the methodology proposed in this paper could be used in any other test cycles such as NEDC (New European Driving Cycle), WLTC (Worldwide Harmonized Light Vehicles Test Procedure), etc. And it has comprehensive application value and are significant for reducing the cost of durability testing of fuel cell systems and promoting the industrialization of fuel cell electric vehicles.

## Introduction

With the increasing importance of the environmental and energy crisis, developing new energy vehicles has become a significant approach for automobile manufacturers^1,2^. The fuel cell vehicle (FCV) has gained recognition for its high efficiency and near-zero emissions, making it an attractive option for future development^3,4^. However, the durability of FCVs is considered a technical challenge and a critical factor for commercialization^5,6^. As a result, significant time and resources have been invested in research related to this area worldwide^7,8^. Currently, the primary fuel cell durability research is based on single cell and fuel cell stack^9,10^. Extensive research has also been conducted on the factors affecting fuel cell stack performance^11–13^. Defining the test cycle is crucial in durability research^14,15^. Currently, two primary methods exist to develop test cycles for analyzing the fuel cell's durability. The first is to design the test cycle based on the factors affecting the fuel cell's longevity. In 2011, Bloom et al. utilized a square wave cycle in dry and wet conditions to simulate idling and full-power conditions in a vehicle^16^. In 2017, IEC also utilized square waves to create a specific test cycle. Unlike USdrive, IEC substituted the idle condition with a 20% power condition^17^. In 2017, Giantleap, a research project backed by the European Union, published its durability test scheme. The main difference is that Giantleap conducted both regular and accelerated durability tests by managing the time and frequency of loading and unloading while retaining the same loading and unloading rates of current density^18^. In 2018, researchers from Wuhan University, led by Tian et al., developed a more elaborate durability test cycle combining open-circuit voltage, idling, variable load, full power, and overload conditions^19^. Moreover, many researchers analyzed the causes of the components performance degradation, such as the electro catalyst and its support^20^, the proton exchange membrane^21^, and the bipolar plate^22^. Meanwhile, some research try to predict the lifetime^23^. This approach has the advantage of being easy to control variables and suitable for testing fuel cells or stacks. However, it does not reflect the actual conditions the fuel cell system may encounter where various control strategies are deployed to prevent damage.

The other method that defines the test cycle based on actual road conditions is developed to compensate for this disadvantage. For example, in 2009, Lin et al. from Tongji University developed a fuel cell stack durability test cycle based on New European Driving Cycle (NEDC) condition. Lin tries to establish the relationship between vehicle speed and fuel cell system current density^24^. In 2013, U.S. Department of Energy (DOE) proposed a durability test cycle based on United States road characteristics^25^. In 2018, researchers from Tsinghua University, led by Xu et al.^26^ developed new durability cycles based on data from actual buses operating on the road. This innovative approach utilizes statistical principles to extract features of the road conditions. However, the new test cycle still has disadvantages, such as limited acceleration and long testing time. Currently, extensive research has been conducted on the durability of fuel cell, from a single cell to fuel cell stack. However, during experiments on a single cell or fuel cell stack, the test bench provides ideal gas pressure, thermal management, and humidity, which differs from real-world vehicle usage scenarios. Therefore, the most rational DUT (device under test) should be a fuel cell system in terms of cost and representativeness. However, there is a lack of durability test cycle at the fuel cell system level.

In this paper, firstly, we propose a model of developing durability test cycle for fuel cell system based on the China automotive test cycle (CATC). By using methods such as frequency reduction processing, smoothing, and power equalization, the initial durability test cycle V_1_ is obtained. Then the test cycle is verified by using a single fuel cell test. To enhance the degradation effect, the durability test cycle V_1_ is optimized by increasing the low-power section ratio and load-changing frequency, resulting in the durability test cycle V_2_. Then, the durability tests for both V_1_ and V_2_ are conducted on the same fuel cell. Finally, a 1000 h V_2_ comparison tests of the fuel cell systems of three mass-produced models are conducted to verify the degradation effect.

## Develop and optimize durability test cycles for fuel cell systems

### Develop and validate the durability test cycle

#### Durability test cycle development

**Figure 1:**
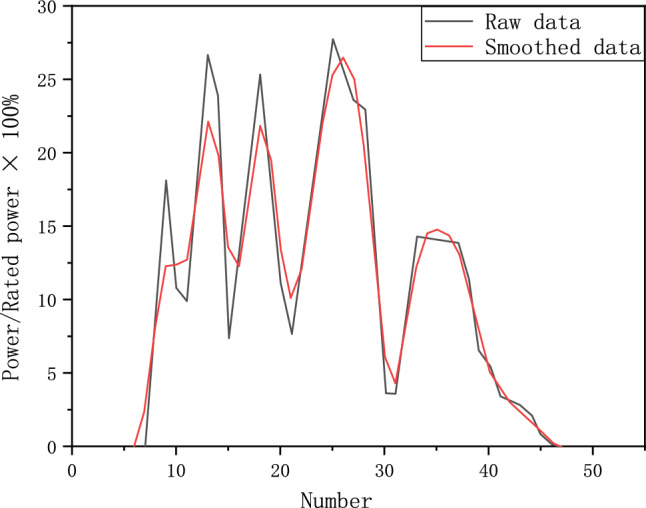
Data smoothing.

China automotive test cycle (CATC) is based on data from 41 cities, 16 million kilometers^27^. CATC incorporates various factors derived from big data, which makes it highly representative. To ensure the durability test cycle of fuel cell system is more representative, power response data of the fuel cell system from different fuel cell electric vehicles are recorded with CATC. Then, the average power is used as the baseline for the fuel cell system. Finally, the data after smoothing was arranged in ascending order, as shown in Fig. 1.

A new method is developed to accelerate the durability effect and simplify the test cycle. The basic principle is replacing the data between a particular range with the average value of all the data within that interval. For instance, the data from 0 to 0.12 kW is replaced by the average value of 0.0312 kW. Through this process, the data could be simplified. For simplification, five equal data segments are used. Each point on the graph represents the same period, and the data for the fuel cell system has been arranged from smallest to largest. This division into five equal segments means that the percentage of time spent in different power segments is the same. This method of processing the data enhances the typicality of the test cycle. Additionally, this paper utilizes a normalization process to convert the actual power of the fuel cell system into a percentage of the rated power. Table 1 shows the results of this process.

**Table 1:** Processing of the data.

Range (kW)	Target value (kW, average of the range)	Power percentage (%)
[0–0.12]	0.0312	10
(0.12–5.615]	2.5694	30
(5.615–14]	8.9814	40
(14–23.444]	17.4171	60
(23.444–75.90]	38.5227	100

**Figure 2:**
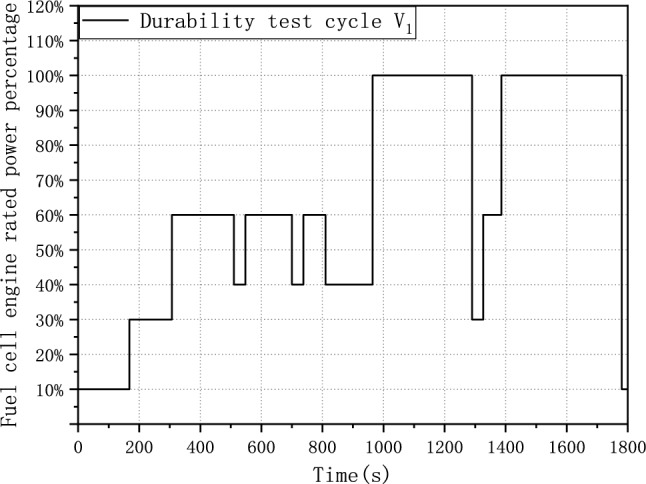
Durability test cycle V_1_.

After simplification and normalization, the durability test cycle V_1_ is presented in Fig. 2. The load and unload rates have been further optimized to ensure the reproducibility.

**Figure 3:**
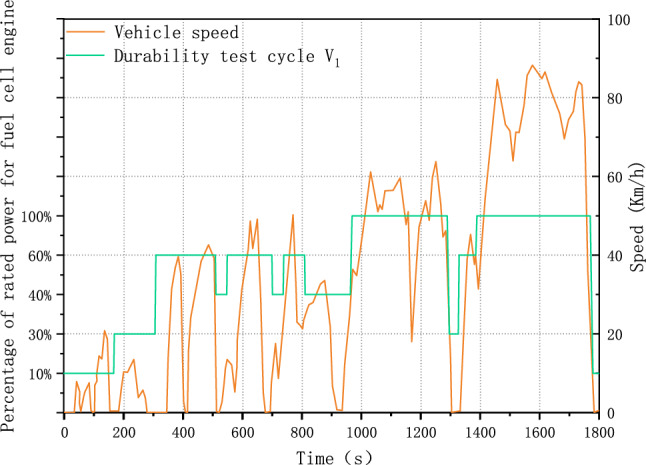
Simplified and normalized durability test cycle.

Figure 3 plots the durability test cycle V_1_ and the power response of the fuel cell system. It shows that the simplified durability test cycle retains the fundamental pattern of power response of the fuel cell system and could accelerate degradation due to a faster load-changing rate.

#### V_1_ validation on single fuel cell

A single-cell validation test is conducted to evaluate the impact of the durability test cycle V_1_. Its specification and test results after the durability test cycle (V_1_) are presented in Table 2.

**Table 2:** Specification and test result of a single cell after V_1_.

Specification	Value
Rated current density (A/cm^2^)	1.2
Initial rated average single cell voltage (V)	0.6741
End rated average single cell voltage (V)	0.6100
Voltage degradation rate (μV/h)	128.2
Cycle operating time (h)	500
Voltage degration percent (%)	9.5

Table 2 shows the specification and result of a single cell and its degradation after V_1_ cycle. The results show that after 500 h of V_1_ cycle, the voltage of single fuel cell experiences a degradation of 9.51%. This indicates that this test cycle could accelerate the fuel cell's degradation. While this durability test cycle may be suitable for some fuel cell systems, it may have limited applicability due to rapid technological progress made in the future. Furthermore, when testing durability at the system level, various strategies could be deployed to prevent fuel cell degradation^28^. Therefore, a method should be developed to optimize V_1_ to meet current and future development needs and to satisfy the desired degradation effect at the system level is necessary.

### Optimize and validate durability test cycle

#### Analyze factors affecting durability

A literature review is conducted to identify factors affecting fuel cell degradation and improve durability. Zhao and X. Li reviewed the steady and accelerated test cycle on the degradation effect and summarized that the steady test cycle resulted in a degradation rate of about 10–20 μV/h. The accelerated test cycle could have a much higher degradation rate of over 200 μV/h^12^. They also find that the square wave condition is the most commonly used accelerated cycle. Moreover, Kneer validates the effect of the frequency and amplitude of square waves on durability. Results show that the voltage drop rate from 0.4 to 0.95 V @ 70 °C is approximately 110 μV/h, while the voltage drop rate from 0.6 to 0.95 V @ 90 °C is around 525 μV/h. Degradation from 0.6 to 0.95 V is much more significant^29^. This suggests that fluctuations in the lower power have a more significant degradation effect on durability. Bae's research on the effects of frequency showed that higher frequency square waves have a more significant impact on durability due to accelerated corrosion of cathode carbon carriers caused by localized starvation of hydrogen at the anode-hydrogen/vacuum interface^23^. This explains why voltage fluctuations in the low-power are more likely to cause degradation, as hydrogen starvation is more likely to occur in this band.

#### Optimize durability effect

**Figure 4:**
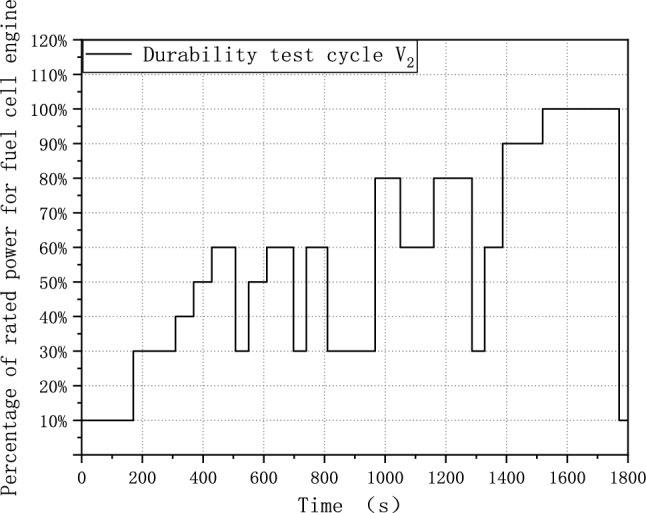
Durability test cycle V_2_.

In the previous section, we analyzed the factors affecting the durability of fuel cells. The main factors affecting fuel cell durability are the low-power section ratio, square wave, and load-changing frequency. However, it is more practical to focus on increasing the low-power section ratio and load-changing frequency for the fuel cell system. Therefore, both increasing the low-power section ratio and load-changing frequency are chosen to optimize the durability test cycle. This could result in a more significant degradation. To achieve this, we have adopted the same development method as V_1_ but modified the segmentation interval of the data from 5 to 9. The optimized durability test cycle is referred to as V_2_ and is plotted in Fig. 4.

**Figure 5:**
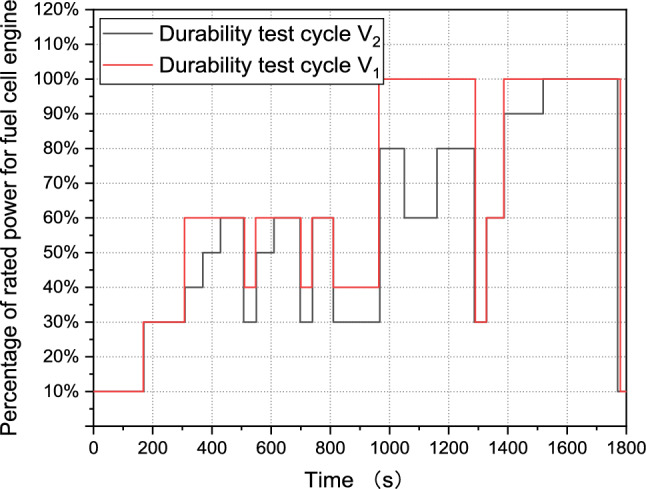
Comparison of Durability test cycle V_2_ and Durability test cycle V_1_.

Figure 5 compares the durability test cycle V_2_ and V_1_. It demonstrates that the optimized test cycle V_2_ has a higher load frequency than the original V_1_. Additionally, the average power is reduced from 65 to 56% rated power. This indicates that increasing the number of data segments from five to nine could increase the load-changing frequency and the proportion of low power.

#### V_2_ validation on single fuel cell

**Figure 6:**
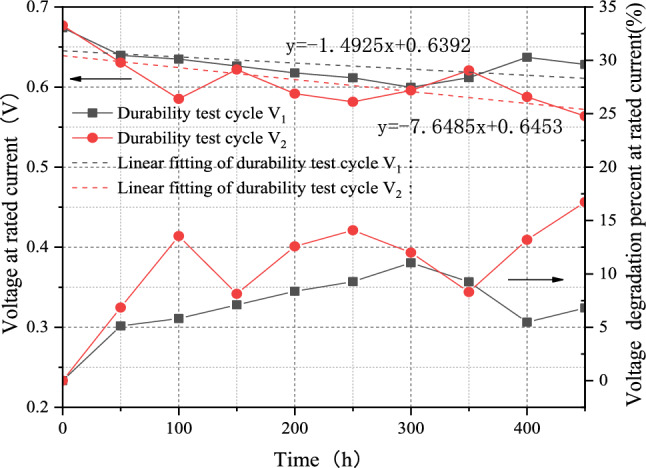
Comparison of degradation effects between V_1_ and V_2_.

To test the degradation effect of the optimized V_2_, a validation test was conducted on the same single cell as in “V_1_ validation on single fuel cell” section. The single cell is tested for 500 h with V_2_, and the voltage at the rated current is measured every 50 h. The voltage degradation curve can be seen in Fig. 6. The linear regression of a single fuel cell with test cycle V_1_ and V_2_ are shown in Eqs. (1) and (2), respectively.

1 $$ {\text{y}} = - 1.4925{\text{x}} + 0.6392 $$

2 $$ {\text{y}} = - 7.6485{\text{x}} + 0.6453 $$

The slope s reflects the overall degradation rate of a single fuel cell, as shown in Fig. 6. The overall voltage degradation rate gradually increased as time passed. However, comparing the voltage degradation rate after linear regression, it is found that the degradation rate of fuel cell using V_2_ is much more than V_1_. The degradation rate was more than five times, and it could reach the U.S. DOE's goal, a decrease of the rated power by more than 10%, more quickly^12^. It is observed that increasing load-changing frequency and low power section can accelerate the degradation effect. The optimized durability test cycle has a more significant degradation effect.

## V_2_ validation on fuel cell system

### Fuel cell system specification

The fuel cell system comprises of fuel cell stack, hydrogen supply and circulation systems, oxygen supply systems, water/heat management systems, control systems, and various components^30^. The degradation of fuel cell system is close to the actual degradation experienced by fuel cell vehicles in the real world. Considering cost and representativeness, the best test subject is a fuel cell system. Experiments on three fuel cell systems from production models were conducted to assess the degradation effect on the fuel cell system. For confidentiality, they are referred to as FCE-A, FCE-B, and FCE-C, with rated power of 60 kW, 86 kW, and 94 kW, respectively. Table 3 shows the parameters for each system. The durability test was conducted based on the V_2_ durability test cycle, as shown in Fig. 5 in “Optimize and validate durability test cycle” section. The cycle time was set at 1000 h, or 2000 cycles, with a polarization curve test interval of 200 h. Through these experiments, we verified the degradation effect of the durability test cycle V_2_ and further evaluated its effect in actual applications.

**Table 3:** Parameters of the test object.

Test object	FCE-A	FCE-B	FCE-C
System rated power (kW)	60	86	94
Cycle operating time (h)	1000	1000	1000
Polarization curve testing interval (h)	200	200	200

### Test results and discussion of fuel cell system durability

#### Voltage degradation of fuel cell system

**Figure 7:**
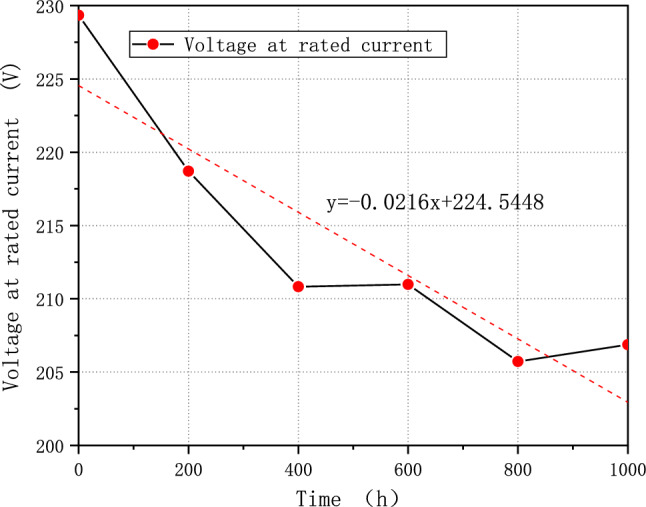
FCE-A voltage at rated current.

**Figure 8:**
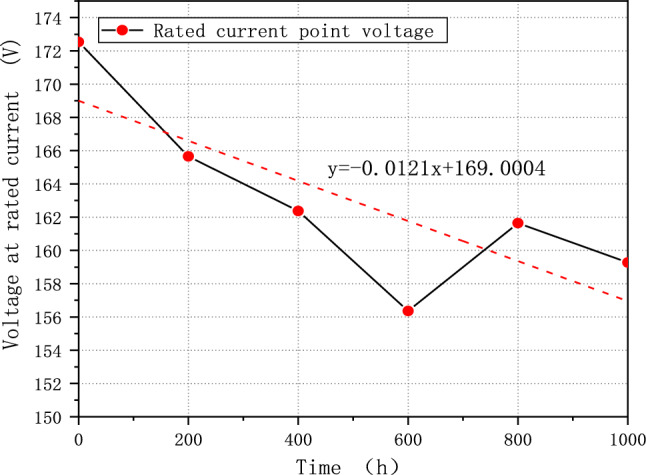
FCE-B rated voltage at rated current.

**Figure 9:**
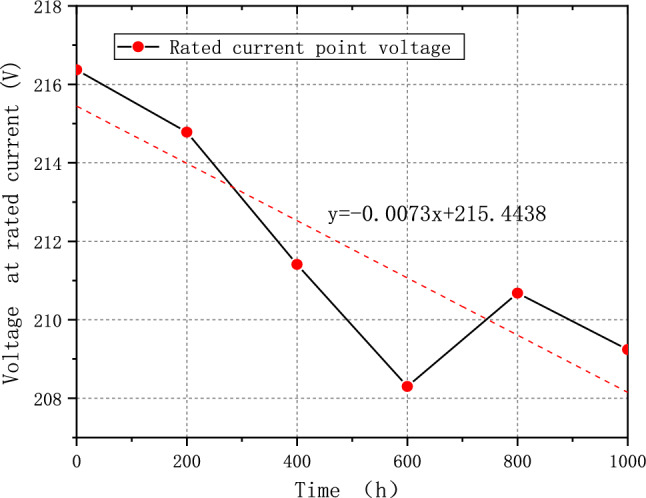
FCE-C voltage at rated current.

The average voltage of the fuel cell system is essential to measure the performance of a fuel system. For example, according to China's demonstration operation policy, the average single cell's voltage should not fall below 0.65 V^31^. After 1000 h of cycling, FCE-A, FCE-B, and FCE-C experience a decay in the average single voltage at rated current by 9.37%, 7.69%, and 3.29%, respectively. The decay rates were 63.87 μV/h, 47.38 μV/h, and 21.60 μV/h, respectively. The degradation pattern is more evident in the linear regression, as shown in Figs. 7, 8, and 9. The linear regression equations are as follows:

3 $${\text{y}}=-0.0216{\text{x}}+224.5448$$

4 $${\text{y}}=-0.0121{\text{x}}+169.0004$$

5 $$y=-0.0073x+215.4438$$

The slope can reflect the overall voltage degradation rate of the fuel cell system. Based on the three figures, it is evident that the voltage of all fuel cell systems initially dropped quickly and then continued to decline before showing a slight recovery^32^. This pattern is exactly like the process of fuel cell polarization. It suggests that the fuel cell system degraded and underwent the typical aging cycle during the durability test with V_2_.

Moreover, fuel cell stack activation is the reason that voltage value corresponding to the rated power of 800 h in Figs. 8 and 9 is higher than the value of 600 h. The water generated during the activation process makes MEA more humid, while increasing the activity of the catalyst and reducing the overall internal resistance of the stack, thus improving the overall performance and stability of PEMFC. However, it also depends on the applied strategy and control ability of manufacture.

#### Power degradation of fuel cell system

**Figure 10:**
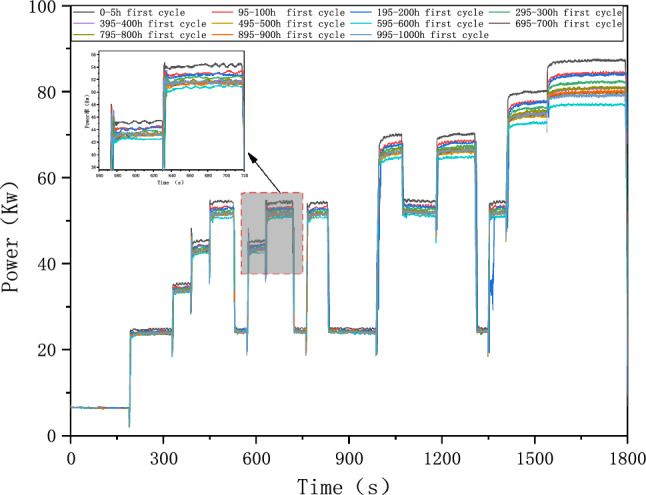
Power degradation of 0-1000h durability test cycle.

The decline of output power is the final result of the fuel cell system's degradation, as plotted in Fig. 10. The figure shows that FCE-B's output power varies as V_2_ cycle. It is observed that the system power decreases overall as the number of cycles increases. The power decline is most evident between the first cycle of 0-5 h and the first cycle of 95–100 h. By the first cycle of 595–600 h, the system output power reaches the lowest. After then, the system output power starts increasing until the first cycle of 795–800 h. This phenomenon corresponds to the fuel cell system's regular operation and validates the durability test cycle's rationality. The other two test cycles exhibit similar trends, which will not be repeated here.

In order to accurately assess the deterioration of the fuel cell system, its output power at the reference current could be calculated using Eq. (6):

6 $$\Delta {P}_{FCE}=\frac{{P}_{0}-{P}_{1}}{{P}_{0}}\times 100\mathrm{\%}$$

where $\Delta {P}_{FCE}$—output Power degradation percent of the fuel cell system at the reference current, in kilowatts (kW); ${P}_{0}$—Power of the fuel cell system at the reference current before the durability test, in kilowatts (kW); ${P}_{1}$—Power of the fuel cell system at the reference current after the durability test, in kilowatts (kW).

**Figure 11:**
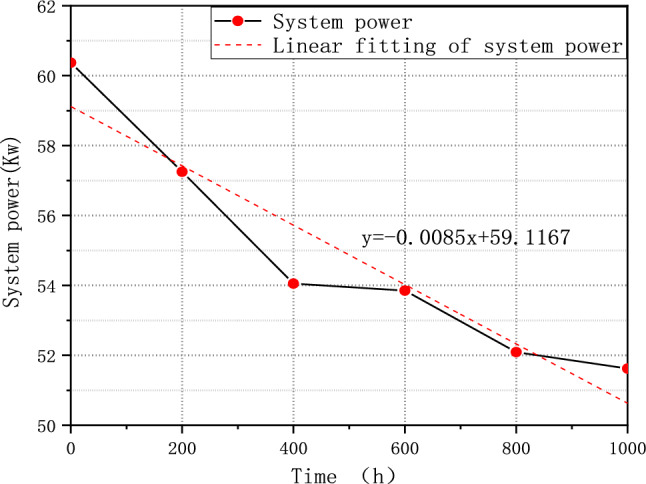
System output power degradation for FCE-A.

**Figure 12:**
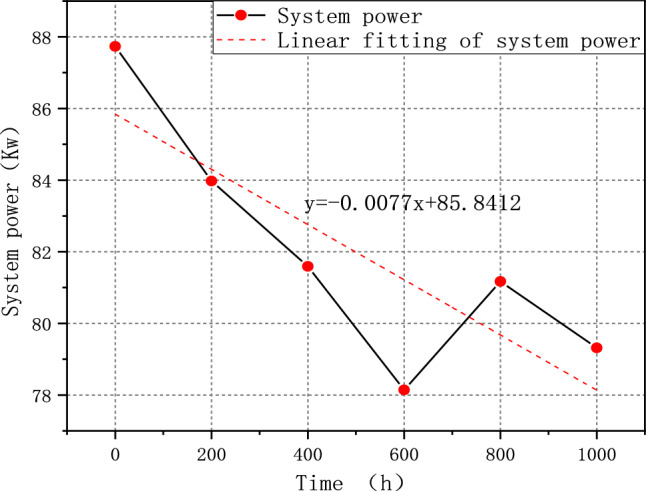
System output power degradation for FCE-B.

**Figure 13:**
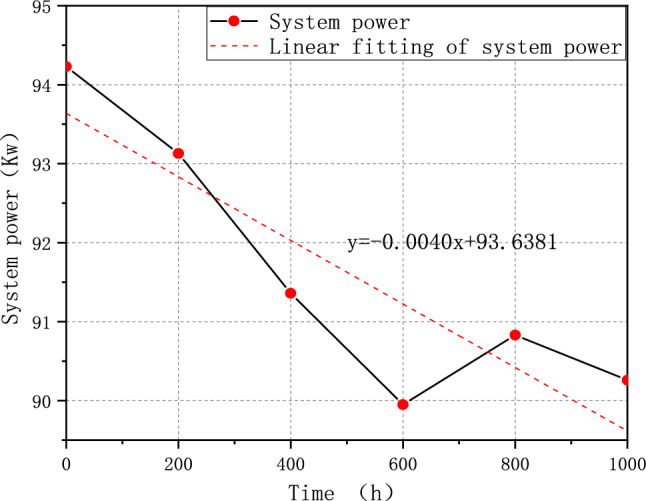
System output power degradation for FCE-C.

The test result shows that FCE-A, FCE-B, and FCE-C degrade by 14.49%, 9.59%, and 4.21%, respectively. After a 1000-h cycle, each fuel cell system shows performance degradation to some degree. It indicates that different fuel cell systems have different deterioration with V_2_. Therefore, the durability test cycle V_2_ can be used to evaluate different fuel cell systems' ability to resist deterioration. The corresponding rated system power of FCE-A, FCE-B, and FCE-C over time is plotted in Figs. 11, 12, and 13. The linear regression equations are as follows:

7 $${\text{y}}=-0.0085{\text{x}}+59.1167$$

8 $${\text{y}}=-0.0077{\text{x}}+85.8412$$

9 $${\text{y}}=-0.0040{\text{x}}+93.6381$$

The slope can reflect the overall output power degradation rate of the fuel cell system.

During the durability test, the fuel cell system's output power have reduced over time, but the percentage of the parasitic power required to keep the fuel cell system running increased. As shown in Table 4.For FCE-A, the parasitic power percent increase from 17.96 to 20.42%. For FCE-B, the parasitic power percent increase from 25.36 to 32.25%. For FCE-A, the parasitic power percent increase from 13.77 to 14.89%.This parasitic power is needed for various components such as water pump, air compressor, hydrogen pump, controller, etc. This increase was mainly due to the fuel cell system's degradation, which increased the energy required to maintain the fuel cell stack running at its rated operating condition. This increase in parasitic power is consistent with the actual degradation of the fuel cell system in the vehicle.

**Table 4:** Percentage of parasitic power in fuel cell systems.

Time (h)	FCE-A (%)	FCE-B	FCE-C
0	17.96	25.36	13.77
200	18.29	26.06	13.88
400	19.35	28.71	14.38
600	19.93	29.29	14.53
800	19.38	30.31	14.81
1000	20.42	32.25	14.89

#### Power consumption of main components

**Figure 14:**
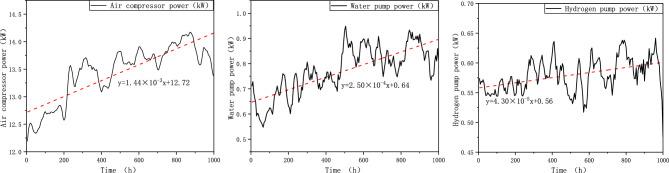
The energy consumed by the air compressor, water pump, and hydrogen pump to maintain the rated power of the FCE-C system change in duration test.

Fuel cell system mainly includes air compressors, hydrogen pumps, water pumps, etc. During the durability testing, these components may experience performance degradation due to wear and failure. As the result, energy consumption of these components will increase at rated power of the system. Figure 14 takes the FCE-C as the example to show the result. The linear regression equations are as follows:

10 $$ {\text{y}} = 1.44 \times 10^{ - 3} {\text{x}} + 12.72 $$

11 $$ {\text{y}} = 2.50 \times 10^{ - 4} {\text{x}} + 0.64 $$

12 $$ {\text{y}} = 4.30 \times 10^{ - 5} {\text{x}} + 0.56 $$

In this case, the degradation of air compressor is obviously comparing to water pump and hydrogen pump, and the degradation rate is comparable to the system power or voltage degradation shown in “Voltage degradation of fuel cell system” and “Power degradation of fuel cell system” sections. This phenomena, 12% degradation, is also shown in Bao’s research^33^. Gemman’s research shows that compressors can degrade in performance over time as rotating components, especially bearings, wear down and surfaces become contaminated with air-borne dirt, dust and oils, and motor windings overheat and fail^34^. The components degradation mechanism is complicated and depend on different factors like material, design, manufacturing, strategy, etc. Quantitative research will be revealed in our future research.

## Conclusions

This paper aims to address the issue of a lack of durability test cycle for fuel cell systems. A universal model is proposed to develop the durability test cycle based on the pattern of fuel cell system in the actual scenario. With optimization and single-cell test verification, the fuel cell system of three mass-production models are compared side-by-side to validate the effectiveness of the durability test cycle. Based on the research, the following conclusions are drawn:

1. This paper proposes a universal model for developing a durability test cycle for fuel cell system based on the China automotive test cycle (CATC). To enhance the degradation effect of the fuel cell vehicle, the durability test cycle V_1_ is optimized by increasing the low-power section ratio and load-changing frequency, resulting in the durability test cycle V_2_. The durability tests for both V_1_ and V_2_ are conducted on the same fuel cell, and the optimized durability test cycle V_2_ results in more than five times degradation than V_1_ at the single cell level. Moreover, the methodology proposed in this paper could be used in any other test cycles such as NEDC (New European Driving Cycle), WLTC (Worldwide Harmonized Light Vehicles Test Procedure), etc.
2. To evaluate the effectiveness of the optimized test cycle V_2_, a 1000 h V_2_ comparison test of the fuel cell systems of three mass-produced models is conducted. The test results show that the three systems' degradation are 14.49%, 9.59%, and 4.21%, respectively. This comprehensive analysis of the degradation of the fuel cell systems confirms that the test cycle developed by the model proposed in this paper can accelerate the durability testing of fuel cell systems and effectively evaluate their durability performance.
3. The durability test cycle development and optimization model proposed in this paper has comprehensive application value and is significant for reducing the cost of durability testing of fuel cell systems and promoting the industrialization of fuel cell electric vehicles.

## Data Availability

The datasets generated and/or analysed during the current study are not publicly available due to the deal with manufacture but are available from the corresponding author on reasonable request.
